# Revolutionizing Lung Cancer Detection: A High‐Accuracy Machine Learning Framework for Early Diagnosis

**DOI:** 10.1155/bmri/9961773

**Published:** 2025-12-12

**Authors:** Tahir Muhammad Ali, Azka Mir, Attique Ur Rehman, Mamoona Humayun, Momina Shaheen, Rafeef Taresh Suliman Alshammari

**Affiliations:** ^1^ Department of Computer Science, Gulf University for Sciences and Technology, Mubarak Al-Abdullah, Kuwait; ^2^ Department of Software Engineering, University of Sialkot, Sialkot, Pakistan, uskt.edu.pk; ^3^ Department of Computing, School of Arts Humanities and Social Sciences, University of Roehampton, London, UK, roehampton.ac.uk; ^4^ Department of Computer Science, College of Computer and Information Sciences, Jouf University, Sakakah, Al Jouf, Saudi Arabia, ju.edu.sa

**Keywords:** classification, literature synthesis, lung cancer, machine learning, prediction model, systematic analysis

## Abstract

Lung cancer is a deadly disease. According to a report of 2024, it is the primary reason for 1.82 million deaths. Given the high disease burden, early detection of lung cancer is crucial for improving survival rates and implementing effective strategies. This paper is aimed at conducting a systematic literature review and developing a highly accurate framework for predicting lung cancer effectively. Tollgate methodology has been used for systematic literature review, and quality assessment criteria were applied to select published articles relevant to the research questions. The paper investigates the effectiveness of machine learning in identifying patterns relevant to lung cancer prediction (Q1), examines the pros and cons of current predictive systems (Q2), compares the use of artificial intelligence in lung cancer prediction with traditional methods (Q3), and identifies key features that distinguish lung cancer from patient symptoms (Q4). Machine learning techniques were employed for the proposed framework. Two publicly available, distinct datasets containing clinical features were obtained. Then, the SelectKBest method was used for feature selection, and SMOTE was used to handle class imbalance. Our proposed framework includes a voting ensemble with random forest, support vector machine, and logistic regression with cross‐validation. The results indicate an accuracy of 99% and 92.5% for the first and second datasets, respectively. This study′s systematic literature review, based on four research questions and a machine learning model, exhibits high accuracy in predicting lung cancer.

## 1. Introduction

**Figure 1:**
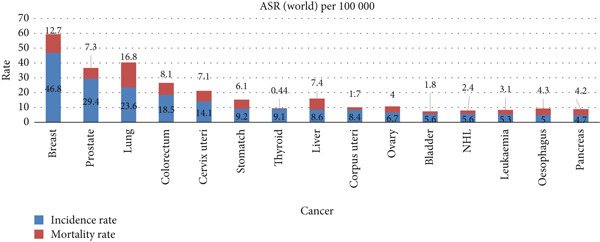
Incidence and mortality rate in 2022.

Lung cancer ranks second in terms of commonly occurring diseases in the world. In 2022, the number of fatalities reached 9.7 million from deaths with different types of cancer, out of which 1.82 million deaths were due to lung cancer alone, making it the most significant contributing factor to death worldwide. According to a report by the World Health Organization (WHO), around 2.5 million new cases diagnosed with lung cancer were observed in 2022 [1]. Therefore, lung cancer is a major public health issue. As of the global estimates from GLOBOCAN 2022 (released February 2024), lung cancer remains one of the leading causes of cancer‐related deaths worldwide [2]. Figure 1 shows that 16.8% of deaths were due to lung cancer, demonstrating the highest incidence rate of the Top 15 types of cancer.

**Figure 2:**
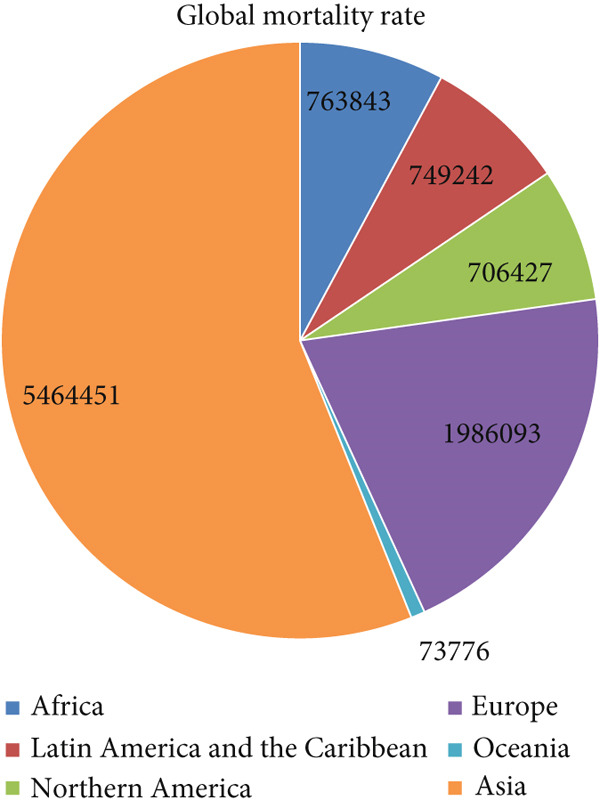
Global estimated number of deaths.

Lung cancer is a prevalent disease with a significant impact on a large number of individuals worldwide. Alongside diagnostic findings, epidemiological parameters such as mortality, incidence, and survival rates play crucial roles in understanding lung cancer [3]. The mortality of lung cancer globally reflects the distribution of deaths attributed to this disease across various demographic groups. According to the Global Cancer Observatory, Asia accounted for 56.21% of lung cancer deaths, Oceania 0.76%, Africa 7.8%, Latin America and the Caribbean 7.7%, Northern America 7.2%, and Europe 20.4% [4]. Figure 2 illustrates the estimated global mortality rates due to lung cancer in 2022.

These records indicate that lung cancer presents a significant health issue. It is classified as a type of cancer that arises from the uncontrolled, abnormal proliferation and growth of cells in the lungs. It is a grave illness that can damage the organs and lead to fatality. Apart from the alarming rise in the incidence rate, there are several other issues encompassing the spread of cancer to other organs (metastasis) and the emergence of complications [5]. Lung cancer exhibits diverse characteristics and is frequently diagnosed at advanced stages, limiting the treatment options [6]. Early detection can have a substantial impact on the survival rate by 20% [7]. Hence, it is imperative to identify it at early stages through the utilization of contemporary methodologies [8].

Machine learning is used for decision support and classification with complex data, enabling models to learn and improve performance. It plays a key role in decision‐making across various fields, including healthcare [9]. In healthcare, machine learning is widely applied, especially for diagnostics [10–12], with professionals increasingly using it to improve diagnostic accuracy and perform predictive analysis, leading to better outcomes [13].

Several machine learning techniques are employed for early lung cancer prediction, using diverse datasets like CT scans, x‐ray images, blood samples, and initial symptoms for clinical diagnosis [14–16]. Current research focuses on utilizing effective classifiers for disease prediction [17–19], with ongoing efforts to enhance outcomes and understand the factors influencing lung cancer prediction and progression. This research is aimed at addressing pertinent research questions that offer valuable insights and findings.

RQ 1. How do machine learning classifiers compare in identifying patterns for predicting lung cancer?

RQ 2. What are the potential benefits and limitations of the existing models?

RQ 3. How does the utilization of artificial intelligence (AI) in predicting lung cancer compare to conventional methods?

RQ 4. What are the key features that can be utilized to predict the likelihood of lung cancer based on a patient′s symptoms?

This research develops a model for accurate lung cancer prediction, presenting a systematic review to address four research questions (RQ 1–RQ 4) using the tollgate methodology. It compares existing models and introduces a framework with clinical datasets. Machine learning techniques drive the high‐performance model, employing SelectKBest for feature selection and synthetic minority oversampling technique (SMOTE) for class imbalance.

**Figure 3:**
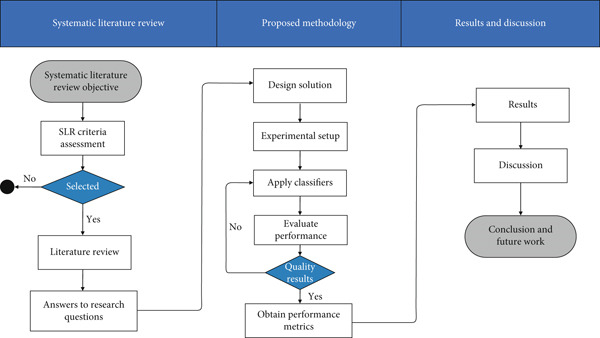
Stepwise organization of the article.

Various classifiers, such as random forest (RF), K‐nearest neighbors (KNNs), support vector machine (SVM) [20], decision tree (DT), naïve Bayes (NB), and logistic regression (LR), are employed during the experimentation phase [21, 22]. The paper is structured as follows: Section 2 details the systematic review methodology and findings, Section 3 outlines the proposed framework and experimental setup, and Section 4 discusses performance evaluation. Section 5 compares systems and answers research questions, while Section 6 concludes with future work, as illustrated in Figure 3.

## 2. Systematic Literature Review (SLR)

**Figure 4:**
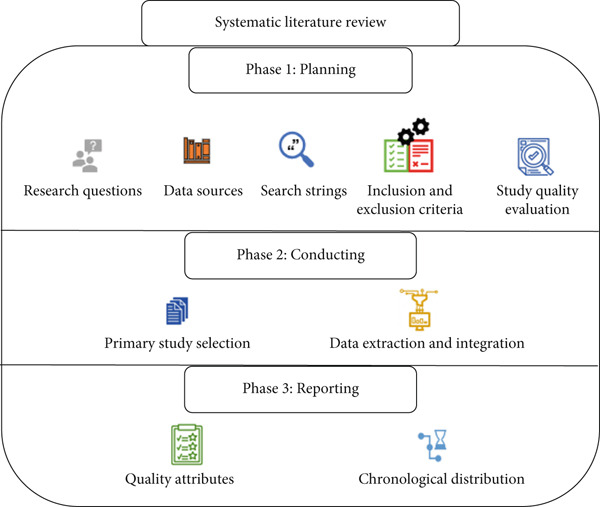
Systematic literature review outline.

A SLR on the use of machine learning for early lung cancer prediction has been conducted. The SLR evaluates existing methods based on inclusion criteria and exclusion criteria to uphold the quality of the studies included. Tollgate methodology has been employed to conduct the SLR, which is used for the identification of the relevant studies [23–25]. Figure 4 shows the outline of a SLR.

### 2.1. Phase 1: Planning the Review

The first phase involves planning the review, which includes identifying relevant, high‐quality studies on machine learning for lung cancer prediction. The following research questions were developed to conduct the review.

RQ 1. How do machine learning classifiers compare in identifying patterns for predicting lung cancer?

RQ 2. What are the potential benefits and limitations of the existing models?

RQ 3. How does the utilization of AI in predicting lung cancer compare to conventional methods?

RQ 4. What are the key features that can be utilized to predict the likelihood of lung cancer based on a patient′s symptoms?

A variety of electronic databases were used to select conference papers and journal publications for this systematic review for diverse perspectives and a comprehensive understanding. The data sources used for the selection of the research work are given in Table 1.

**Table 1:** Data sources, filters, and search scope used in systematic review.

Electronic database	IEEEXplore, MDPI, Elsevier, Hindawi, PubMed, Springer Link, Nature, and Google Scholar
Search resources	Conferences and journal publications
Search applied on	Search is applied on a machine learning application on the disease and excludes the results that do not contain the terms in the title or abstract
Language	English
Publication period	January 2018–2024
Search mechanism	The search mechanism was modified according to the selected database

Search strings were formulated using terms from the titles of existing studies on machine learning in lung cancer. The search terms and repositories used in this paper are given in Table 2.

**Table 2:** Search strings used to obtain existing studies.

**Search strings**	**Repositories**
“Lung‐Cancer prediction machine‐learning”, “Lung‐cancer prediction deep‐learning”	IEEE
“Lung‐cancer prediction”, “lung‐cancer machine learning”	Springer Link
“lung‐cancer prediction” OR “lung‐cancer diagnosis” OR “lung‐cancer prevalence” AND “machine‐learning”	Hindawi
“lung ‐cancer prediction” OR “lung cancer diagnosis” OR “lung cancer prevalence” AND “machine‐learning”	PubMed
“lung‐cancer prediction” OR “lung‐cancer diagnosis” OR “lung ‐cancer prevalence” AND “machine‐learning”	Nature
“lung‐cancer prediction using machine‐ learning”	Google Scholar
“lung‐cancer prediction” OR “lung‐cancer diagnosis” AND “machine ‐learning”	MDPI
Lung‐cancer prediction, lung‐cancer machine learning	Elsevier

Inclusion criteria were developed to narrow the literature, while exclusion criteria filtered out irrelevant studies, as shown in Table 3.

**Table 3:** Criteria for inclusion and exclusion for the filtration of research studies.

**Inclusion**	**Exclusion**
The paper must be featured in a scholarly publication or presented at a conference.	A paper that does not focus primarily on lung cancer prediction using machine learning.
Papers should be related to machine learning prediction of lung cancer.	If more than one version of a paper is available, only the latest and complete version of the paper will be included.
The paper contains the selected search terms in the title.	Studies published before 2018.
The paper should be published between 2018 and 2024.	Non‐English publications.
Relevant papers.	Irrelevant papers.

A checklist to evaluate the quality of the literature was developed using quality evaluation (QE) scores. The quality assessment checklist is given in Table 4.

**Table 4:** Quality evaluation scores based on evaluation criteria.

**QE**	**Evaluation criteria**
QES‐1	Articles with the answer are assigned “1” score.
QES‐2	Articles with partial answers are assigned “0.5” score.
QES‐3	Articles with no answers are assigned “0” score.
QE‐questions	Quality evaluation checklist for questions
QE. 1	Does the methodology contain the answer to the research question?
QE. 2	Do the articles discuss optimal features for selection?
QE. 3	Do the findings contain answers to the research question?
QE. 4	Was the study design relevant and appropriate for answering the research question?

### 2.2. Phase 2: Conducting the Review

After planning, a comprehensive review was conducted in two steps: primary selection based on defined criteria and data synthesis and extraction. The process is given in detail in the following subsections.

#### 2.2.1. Primary Study Selection

A total of 130 research papers were selected from electronic databases by employing the search strings provided in Table 5. The tollgate approach, a five‐phase process, was applied to ensure reliability, quality, and reduce bias in the review. Using criteria for inclusion and exclusion, articles were further narrowed down in each phase, resulting in 40 in the final phase.

**Table 5:** Tollgate method is used to refine the number of selected research papers.

**Digital database**	**Phase 1**	**Phase 2**	**Phase 3**	**Phase 4**	**Phase 5**	**Total (%)**
Elsevier	18	10	3	3	3	8
Hindawi	3	3	3	1	1	2
IEEE	29	20	16	16	16	40
MDPI	10	11	3	2	2	5
Nature	7	2	2	2	2	5
PubMed	10	10	10	2	1	2
Springer Link	16	9	8	6	5	13
Google Scholar	37	27	14	13	10	25
Total	130	85	59	45	40	100

**Figure 5:**
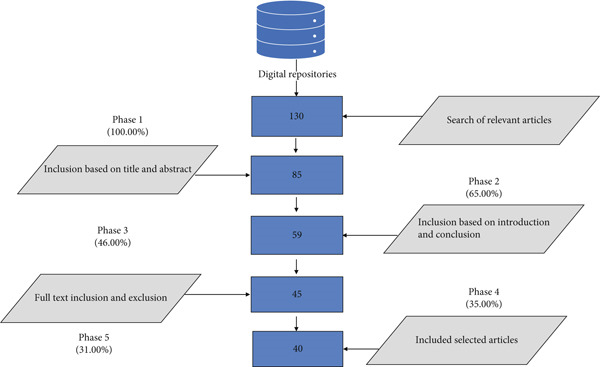
Tollgate method for article selection.

A complete breakdown of the tollgate approach is shown in Figure 5.

#### 2.2.2. Data Extraction and Integration

Data extraction and synthesis are essential for a systematic review, allowing unbiased analysis. Selected papers are evaluated based on research questions, with key aspects of methods, techniques, and findings summarized. The extracted data includes the following:

• Title of paper
• Publication year
• Research methodology
• Repository

### 2.3. Phase 3: Reporting the Review

This phase presents key findings and a summary of the selected studies, ensuring relevance, transparency, and integrity. It includes two subprocesses: quality assessment and temporal distribution. The quality of the papers is assessed with a QE score, and the studies′ temporal distribution spans from 2018 to 2024.

### 2.4. Literature Review

This literature review is aimed at exploring the selected research work according to the criteria. This comprehensive review will examine the incredible capability of machine learning for the prediction of lung cancer while shedding light on areas highlighting the research questions.

An interdisciplinary method for the detection of lung cancer is used by [26]. It introduced a combination of six metabolic biomarkers to enable the discrimination between lung cancer Stage 1 patients and nonpatients. The dataset is obtained from Hubei‐Taihe Hospital, which included 110 individuals with cancer and 43 individuals without cancer. The data used consists only of metabolic levels. The proposed solution uses metabolomics and machine learning methods. The results are AUC = 0.989, 98.1% sensitivity, and 100.0% specificity. A deep learning model for surface‐enhanced Raman spectroscopy (SERS) of the exosomes for the prediction of lung cancer was proposed in [27]. The dataset consisted of 20 healthy plasma samples and 43 lung adenocarcinoma samples. The model classified plasma exosome signals using ResNet, SVM, PCA, LDR, and PLS‐DA, achieving 95% accuracy and AUCs of 0.912 for the whole cohort and 0.910 for Stage 1 cancer. A study on predicting lung cancer using blood indices from 277 patients [28] found that XGBoost, using GridSearchCV, outperformed other models with sensitivity of 96.67%, a specificity of 85.71%, and accuracy of 92.16%. A survival prediction model for non–small cell lung cancer using data from 3714 patients [29] applied nine machine learning algorithms, with the artificial neural network (ANN) model providing the highest AUC (0.89), 82% accuracy, and 91% precision. The detection of lung cancer using CT scan images was proposed in [30] by applying texture features and statistical feature extraction with seven machine learning classifiers. The dataset contains a total of 15,750 medical images of benign and malignant tumors. Texture features and statistical features were extracted. The accuracy obtained by the proposed system is 88.55% with a multilayer perceptron classifier. An algorithm to identify the cancerous images is presented in [31] using textual features from ROIs by applying a multiclass SVM classifier. The dataset comprises 500 infected and typical lung CT images each. The proposed method uses multistage classification built with MATLAB using an image processing technique. The proposed algorithm provides a precision rate of 97% for identification and a precision of 87%. Lung cancer detection was proposed in [32], utilizing a dataset of 50 low‐dose whole lung CT scans and radiologist‐identified nodule locations. After feature extraction and dimension reduction via LDR, the features were optimized using a modified gravitational search algorithm for classification with a DNN classifier, achieving 94.56% accuracy, 96.2% sensitivity, and 94.2% specificity. A multivariate UCI dataset was used in [33]. The dataset contains 600 instances with three classes, that is, malignant, benign, and premalignant. It was preprocessed with 21 attributes and reduced using PCA. SVM classification resulted in a recall, precision, and *F*1 measure of 0.88. The RF algorithm with convolutional neural network (CNN)–based technique for the prediction of lung cancer using CT scan images was presented in [34]. Two online datasets containing 3954 images and 50 CT scan images of low‐dose whole lungs were used. Feature extraction, feature selection, and then detection of the cancerous spots were performed. It achieved 93.25% accuracy and *F*1 measure = 91.75*%*. A machine learning model on the Kaggle dataset of 15,000 was presented in [35], out of which 1200 images were selected. The dataset is classified into three classes. Feature extraction using Sobel, Gabor, and Gaussian filters followed by RF, KNN, and SVM classification resulted in 84.2% accuracy with RF. An algorithm to track tumors for automated radiation therapy for cancer treatment with graphics processing unit computing was proposed in [36]. The dataset was obtained using a 3T MRI scanner from six patients with free breathing. Thirty images were taken before treatment and 100 during the treatment stage. The study proposes a cost‐reducing parallel GPU platform to speed up the nonrigid image registration algorithm and achieved a 0.87 dice score on 600 image data obtained from six patients.

A system for detecting using CT images based on image‐processing techniques was proposed in [37]. The dataset, from the Cancer Imaging Archive (TCIA), included CT images of cancerous and noncancerous patients. Noise was removed using median filtering, and segmentation was done with morphological operations. Geometrical features (perimeter, area, and eccentricity) were extracted and used as inputs for SVM classification. In [38], data mining techniques and machine learning classifiers were used on a UCI dataset of CT images from 1397 patients, with additional data from 198 patients. Nodules in the CT images were detected, and patches were extracted and encoded. Features were trained, and the model achieved an accuracy of 0.794 with SVM, 0.953 with DT, and 0.945 with KNN. An interpretable system was proposed in [39] using machine learning classifiers on the Kaggle dataset. SMOTE sampling addressed class imbalance, and LR and RF algorithms achieved 97% accuracy. SHAP and LIME were used for explainability, identifying significant features like difficulty swallowing, alcohol consumption, and cough (SHAP) and chronic disease, allergy, and difficulty swallowing (LIME).

A systematic approach for classification with eight classifiers was presented in [40] using the Kaggle dataset. It consists of four predictions and one result attribute with a total record of 59. Among all the classifiers, NB performed with the highest accuracy of 98.33%. A hybrid method using machine learning is proposed in [41] on the Kaggle dataset, which consists of 284 instances of 16 attributes. The work incorporates nonlinear regression (NLR) and Gaussian mixture model (GMM) termed NLR‐GMM algorithm. The hybrid machine learning model achieved 92.88% accuracy. The prediction of lung cancer using linear regression, logarithmic regression, LR, multiple regression, and exponential regression is presented in [42] on the Kaggle dataset. With regression analysis, it is concluded that cough, wheezing, chronic disease, alcohol consumption, and allergy are the significant symptoms that influence lung cancer. For the prediction of lung cancer, an accuracy rate of 96% was obtained by a multiple regression algorithm. An effective classification of lung cancer is presented in [43], which depicts physiological factors on the Kaggle dataset. The experiment uses NB, SVM, KNN, adaptive boosting, J48, and LR. The results show that LR has the highest accuracy and *F*1 measure of 94.7%. A machine learning model for lung cancer classification has been developed in [44] on the Kaggle dataset. The experiment used KNN, SVM, NB, and narrow neural network (NNN) algorithms. SVM provided the highest accuracy of 92.6%.

Machine learning classifiers are applied in [45] to predict lung cancer on the UCI dataset. SVM, KNN, and CNN applied via Weka tool achieved 95.56% accuracy with SVM. Ensemble algorithms in [46] on the same dataset utilized SMOTE with XGBoost, bagging, LightGBM, and AdaBoosting, achieving 94.42% accuracy, 95.66% precision, and 94.46% recall via XGBoost. A CRISP‐DM‐based model in [47] used RapidMiner to identify lung cancer causes, such as smoking and chest pain, with ANN attaining 93% accuracy, 90% specificity, and 91% precision. Classifier efficiency was assessed in [48] using UCI data and patient symptoms, with gradient‐boosted trees achieving 90% accuracy. The prevalence of lung cancer is suggested to have a positive correlation with the proportion of chain smokers in [49] indicating that smoking is a major risk factor. It uses the UCI dataset for classification and achieved 96.9% accuracy by LR with cross‐validation with *k*‐fold 7.

To predict lung cancer, five distinct algorithms have been applied in [50]. The dataset is obtained from the UCI repository. Bayesian network, LR, J48, RF, and NB are utilized, and the outcome was estimated using the evaluation methods and Weka tool. LR performed the best with 91.90% accuracy, NB with 90.29% accuracy, Bayesian network with 88.34% accuracy, J48 with 86.08% accuracy, and RF with 90.93% accuracy. An efficient model to identify the people at risk of developing long‐term complications is presented in [51]. The proposed model uses rotation forest, which provided the best outcomes of AUC = 99.3*%*, *F*1 score, precision, 97.1% recall, and accuracy. A machine learning classification model using a 32 × 56 dataset from UCI [52] focuses on preprocessing with nine datasets and six classifiers (KNN, RF, NB, LR, DT, and SVM), achieving the highest accuracy with KNN. *Z*‐score normalization, PCA for dimension reduction, and information gain for feature selection yielded accuracies of 83%, 87%, and 71%, respectively. For lung cancer prediction using the Kaggle dataset [53], random oversampling (ROS) handles imbalanced datasets, and SHAP selects features. PCA, ROS, and hyperparameter tuning are used with ensemble classifiers (GBM, LightGBM, and XGBoost), where GBM achieves 98.76% accuracy. In a retrospective cohort study [54], a clinical prediction model is proposed for nonsmokers with lung nodules. Analyses were done by binary LR, and their nomogram achieved a strong discriminative accuracy of 75%. The HUNT study in Norway [55], tracking 583 lung cancer cases over 15.2 years, identified key risk factors through Cox regression. A validated model tested on 45,341 ever‐smokers achieved a concordance index of 0.879 and an AUC of 0.87 over 6 years. A systematic review for early lung cancer prediction [56] included 39 studies, showing sensitivity and specificity at 0.87. The review highlights the lack of standardized screening protocols, suggesting that large‐scale validation could improve clinical integration.

Machine learning methods were used to predict 5‐year lung cancer survival based on health‐related quality of life (HRQOL) data [57]. The dataset includes 809 lung cancer surgery survivors, with two feature sets: one with clinical and sociodemographic data and the other adding HRQOL factors. Machine learning models (DT, LR, bagging, RF, and AdaBoost) with fivefold cross‐validation were applied. AdaBoost and RF in Feature Set 2 yielded the best AUCs: 0.850 for DT, 0.898 for LR, 0.981 for bagging, and 0.949 for RF. In [58], machine learning classifiers were applied to a UCI dataset after preprocessing and binary conversion. Using the Weka tool, the model achieved 81.25% accuracy. An overview of AI in cancer detection [59] highlighted its potential benefits for healthcare but noted challenges in large‐scale implementation. Among several algorithms compared, SVM with an RBF kernel achieved the highest accuracy of 95.12%. A machine learning model for predicting ICU length of stay (LOS) in lung cancer patients, using the MIMIC‐III dataset and electronic health records (EHR), is presented in [60]. The model, using RF, identifies key features using SMOTE and SHAP for explainability. SMOTE and ADASYN achieved AUCs of 98% and 100%, respectively, while combined oversampling–undersampling methods (SMOTE‐Tomek and SMOTEENN) showed the second‐highest AUCs. Undersampling methods (ENN and Tomek Links) performed the worst. A study [61] used Bayes‐Net, NB, DT, RF, and an ANN to predict lung cancer based on a Kaggle dataset of 309 instances and 16 attributes. The ANN, with one hidden layer, achieved the highest accuracy of 92.23%. Future work suggested feature selection, addressing class imbalance, and ensemble methods. The study in [62] discusses lung cancer causes, side effects, death rates, and the use of AI in diagnosis and prediction. It focuses on how machine learning, particularly deep learning, has been utilized by specialists to improve the accuracy of prediction and diagnosis in healthcare. To predict cancer [63], machine learning models were used, with the lung cancer dataset containing 1001 rows and 25 features. KNN achieved 97% accuracy in classifying risk levels (low, medium, and high). The dataset [64], from the LIDC/IDRI database, contains 9496 images. After feature extraction and augmentation, the model split data 80% for training and 20% for testing. To predict lung cancer, DT, LR, KNN, SVM, and NB were used for categorization and comparison. SVM achieved 98% accuracy [65].

There was a notable diversity in dataset modalities and algorithmic approaches, yet several methodological patterns and limitations exist. Most works employed relatively small, often domain‐specific datasets, frequently without clear statements on preprocessing and imbalance handling, which raises concerns about generalizability. Classical machine learning methods such as RF, SVM, and LR remain common, though deep learning approaches, particularly CNN‐based architectures, dominate in image‐driven studies. Feature selection methods, when reported, ranged from filter‐based statistical techniques to wrapper methods, but a significant fraction of studies did not address the issue. This thorough review contributes to gaining a comprehensive understanding of the existing work and the identification of gaps in the existing studies. The summary is provided in Table 6.

**Table 6:** Summary of literature review.

**Sr.**	**Authors**	**Year**	**Dataset**	**Feature selection method**	**Algorithm(s) applied**	**Validation method**
1	Xie et al.	2021	Exosomal miRNA profiles (spectroscopic data)	—	ML methods	Cross‐validation
2	Singh and Gupta	2019	Biomarkers	Combination of six metabolic biomarkers	Various ML classifiers	Train‐test split
3	Alam et al.	2018	MRI/CT imaging dataset	—	Multiclass SVM classifier	—
4	Lakshmanaprabu et al.	2019	CT images	—	Deep learning model	—
5	Shin et al.	2020	Spectroscopic exosome data	Likely feature extraction	Deep learning model	Cross‐validation
6	Manju et al.	2021	Clinical/imaging dataset	—	SVM classifier	—
7	Dutta	2022	Imaging/clinical data	—	ML algorithms (general)	—
8	Gupta et al.	—		—	ML algorithms	—
9	Tahmasebi et al.	2020	MRI	—	CUDA no_igid registration	Real‐time tracking validation
10	Nadkarni and Borkar	2019	CT images	—	Image processing (thresholding, etc.)	—
11	Ahmed et al.	2019	Multidimensional data	—	Data mining + supervised ML algorithms	—
12	Ahmed et al.	2023	Clinical/imaging data	—	XAI methods	—
13	Sachdeva et al.	2022	Imaging/clinical	—	Systematic method for classification	—
14	Rajaguru et al.	2022	Symptom‐based clinical dataset	—	Gaussian mixture model + hybrid ML	—
15	Sundar et al.	2023	Clinical dataset	—	Advanced intelligent computing method	—
16	Ojha	2023	Clinical/imaging dataset	—	ML classifiers (general)	—
17	Al‐Tawalbeh et al.	2022	Imaging/clinical	—	Multiple ML algorithms	—
18	Qubahan Journal article	2023	Clinical dataset	Correlation selection	ML algorithms	—
19	Mamun et al.	2022	—	—	Ensemble learning techniques	—
20	Vieira et al.	2021	Lung cancer case data	—	Data mining	—
21	Faisal et al.	2018	—	—	Various ML classifiers and ensembles	—
22	Radhika et al.	2019	—	—	Comparative study of ML algorithms	—
23	Viji Cripsy and Divya	2023	—	Feature selection method	Bayesian network, logistic regression, J48, random forest, and naïve Bayes	—
24	Dritsas and Trigka	2022	—	—	ML models	—
25	Göltepe	2021	—	—	Different classification algorithms	—
26	Rikta et al.	2023	XML‐GBM lung dataset	—	Explainable ML (GBM‐based)	—
27	Zhang et al.	2022	—	—	ML algorithms	Accuracy: 75%
28	Markaki et al.	2018	HUNT study (clinical cohort of smokers)	—	Clinical risk prediction model	Validation cohort
29	Kanan et al.	2024	Multiple datasets (systematic review)	—	Multiple AI‐driven models (review and meta‐analysis)	—
30	Hsu et al.	2022	Multiple data types (clinical and genomic)	—	Deep learning models	—
31	Puneet and Chauhan	2020	Routine blood index dataset	—	ML techniques	—
32	Sim et al.	2020	Lung cancer survivors cohort	—	ML models	—
33	Patra	2020	—	—	ML classifier	—
34	Patel et al.	2019	—	—	AI techniques for cancer detection	—
35	Sarker	2021	—	—	ML algorithms for predictive modeling	—
36	Salama and Ragab	2022	Medical imaging datasets	—	Deep learning models	—
37	Bhatia and Arora	2021	Lung cancer gene expression data	—	Feature selection + classification models	—
38	Li et al.	2021	—	—	ML algorithms	—
39	Islam et al.	2020	Chest CT scan dataset	—	CNN models	—
40	Ben Hassen et al.	2020	Lung nodule dataset	—	Deep learning and hybrid models	—

### 2.5. Analysis of How the Research Questions Are Addressed in the Literature

In light of the comprehensive literature review on the application of machine learning to lung cancer, research questions are taken into account. Answers to research questions are given as follows:

RQ 1. How do machine learning classifiers compare in identifying patterns for predicting lung cancer?

Machine learning classifiers offer several ways to track the patterns found in lung cancer patients to accurately predict lung cancer at early stages. Because different classifiers have different patterns and approaches to analyzing data, they may perform differently in terms of accuracy, interpretability, and computational efficiency. The performance of a classifier depends on several parameters, such as the type of dataset, the size of the dataset, and the features being used. Evaluating multiple classifiers and the comparative analysis of their performance helps to determine the most effective classifier for lung cancer detection. This is the primary reason for applying algorithms to identify the best performing classifiers for a particular dataset. However, existing systems have used various best performing classifiers on clinical features. Table 7 presents the algorithms that were most often utilized in the proposed solution of existing studies.

**Table 7:** Frequently used algorithms.

**Algorithms**	**Count**
K‐nearest neighbors	2
Naïve Bayes	3
Random forest	4
Support vector machine	9
Rotation forest	2
Convolutional neural network	2
Logistics regression	4
Total	26

Classifiers used only once were excluded for brevity and focus on common trends, resulting in a total count of 26 repeated classifier uses. Out of 40 studies, other classifiers are often combined in hybrid models, while ensemble techniques and deep learning classifiers like ANNs and CNNs are gaining traction with larger datasets.

RQ 2. What are the potential benefits and limitations of the existing models?

**Figure 6:**
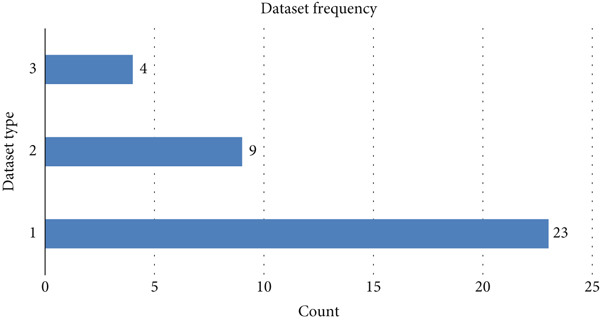
Types of datasets based on usage.

Existing models have shown promise in enhancing early detection, leading to improved treatment outcomes. These models help professionals identify and prioritize at‐risk patients, minimizing costs and optimizing survival rates. The literature review indicates that current systems achieve solid performance metrics. However, challenges remain in lung cancer research, including high false positive (FP) and false negative (FN) rates that can lead to unnecessary procedures or missed diagnoses. Issues like limited access, insufficient data, geospatial limitations, and FPs also affect dataset reliability. The type of datasets used in the existing literature is shown in Figure 6.

This shows that the most frequently used dataset type is based on clinical symptoms. The second highest is CT/MRI‐based dataset. Omics data encompassed blood specimen analysis (*n* = 3), metabolic biomarker profiles (*n* = 1), and cancer cell–derived features (*n* = 1). The type of dataset may have its own flaws; for example, ionizing radiation on CT scan or PET scan may kill healthy cells or disrupt part of the scan. Given these limitations, there are many opportunities to be explored with the current progress. The models could be further improved and integrated with other features for improved performance in the detection of lung cancer.

RQ 3. How does the utilization of AI in predicting lung cancer compare to conventional methods?

AI models have shown some promising results in the quick and accurate analysis of large medical data. The methods used in existing literature have been the cornerstone of lung cancer detection. The findings from the existing studies have highlighted the capability of AI to enhance traditional methods and improve lung cancer detection. AI models have proven to be of great help to professionals. However, AI‐based models should be developed by keeping the limitations under consideration to provide a mutually beneficial relationship between AI and traditional practices. The systematic review of existing research suggests that AI models complement traditional practices by providing additional insights and enhancing the overall diagnostic process.

RQ 4. What are the key features that can be utilized to predict the likelihood of lung cancer based on a patient′s symptoms?

**Figure 7:**
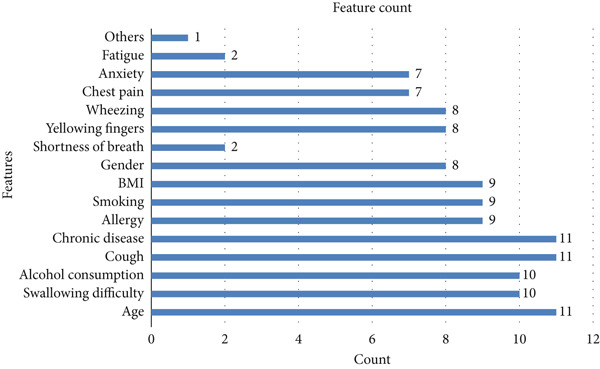
Frequently used features.

The results for the prediction of lung cancer may vary based on different features. The variation may occur due to factors that directly affect the prediction performance. This holds not only for symptom‐based numerical datasets but also for various other datasets such as blood samples, CT images, and biomarkers. However, the early signs may include different machine learning methods applied for feature selection. For instance, difficulty in swallowing, alcohol consumption, and cough were obtained by SHAP; on the other hand, chronic disease, allergy, swallowing difficulty, and alcohol consumption were obtained by LIME. Figure 7 shows the frequently used features in the existing literature.

Frequency shows that the most used features were age, chronic disease, and cough, with a frequency count of 11, with other modalities showing unique features listed with a count of 1 for completeness. While frequency alone may not determine the significance of features, it can still provide valuable insights. These frequently used attributes suggest that they are still noteworthy in the prediction of lung cancer.

## 3. Proposed Solution

**Figure 8:**
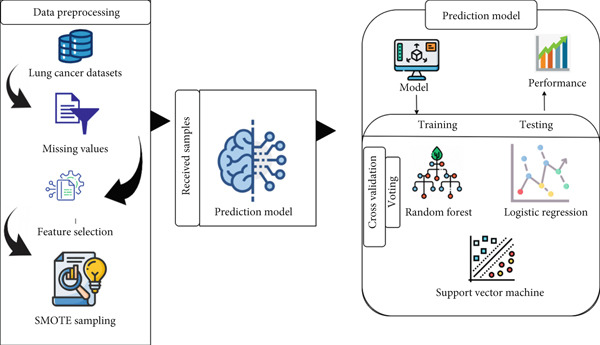
Proposed framework for lung cancer prediction.

This section proposes an innovative framework for accurately classifying lung cancer using machine learning. The solution aims to support early diagnosis via symptom‐based triage. The process involves several stages, from data collection to prediction using statistical methods on both training and testing sets [66]. This framework, with its components, provides a valuable tool for medical decision‐making [67]. The datasets are preprocessed, split into training and testing sets, and tested on algorithms to evaluate performance [68]. The detailed framework is given in Figure 8.

Experiments were conducted using Python, with the classifiers and techniques detailed in the following section. All code was developed and tested using Python 3.10.11, with key libraries including scikit‐learn 1.2.1 and numpy 1.26.4.

### 3.1. Data Collection

The first dataset used in this research is the “cancer patient dataset” obtained from Kaggle [69]. It has 1000 instances and 25 attributes. The target class, labeled as “level”, consists of three categories: low, medium, and high. Table 8 presents a detailed description of the dataset.

**Table 8:** Lung cancer dataset description.

**Features**	**Description**
Patient′s ID	Patient′s ID
Patient′s age	Patient′s age
Patient′s gender	Patient′s gender
Air pollution	Exposure to pollution
Alcohol use	Alcohol use/consumption
Allergy	Dust intolerance
Workplace hazards	Patient′s work‐related risks
Genetic risk	Patient′s genetic risk
Chronic disorder	Chronic disorder level
Balanced diet	Patient′s diet
Obesity	Obesity level
Smoking	Smoking status
Passive smoking	Passive smoking
Chest pain	Chest pain level
Blood cough	Blood in cough
Losing weight	Weight loss
Exhaustion	Fatigue
Difficulty in breathing	Shortness of breath
Wheeze	Whistling sound when breathing
Difficulty in swallowing	Difficulty in swallowing
Clubbing of fingernails	Clubbing of nails
Frequent cold	Frequency of getting cold
Dry cough	Dry cough
Snoring	Snoring
Level	High, low, and medium: Lung cancer level

**Figure 9:**
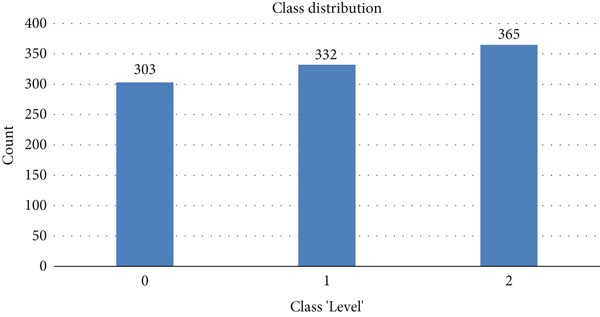
Class “level” count.

The target label “level” is given in Figure 9, which shows the class distribution. The class count presents 303 instances of Level 0, 332 of Level 1, and 365 of Level 2.

The second dataset, “survey lung cancer,” has also been collected from Kaggle [70]. It has 309 instances and 16 attributes. The target feature is “LUNG_CANCER,” which depicts the existence (yes) or absence (no) of lung cancer. Table 9 presents a detailed overview of the dataset.

**Table 9:** Dataset description for lung cancer.

**Features**	**Description**
Gender	Patient′s gender
Age	Patient′s age
Smoking	Smoking level
Yellow fingers	Yellowing of fingers
Anxiety	Presence of anxiety
Peer pressure	Presence of peer pressure
Chronic disease	Presence of chronic disease
Fatigue	Presence of fatigue
Allergy	Presence of allergy
Wheezing	Wheezing
Alcohol consumption	Consumption of alcohol
Cough	Cough
Shortness of breath	Difficulty in breathing
Difficulty in swallowing	Difficulty in swallowing
Chest pain	Level of chest pain
Lung cancer	1 present or 0 absent
Wheezing	Wheezing
Alcohol consumption	Consumption of alcohol

**Figure 10:**
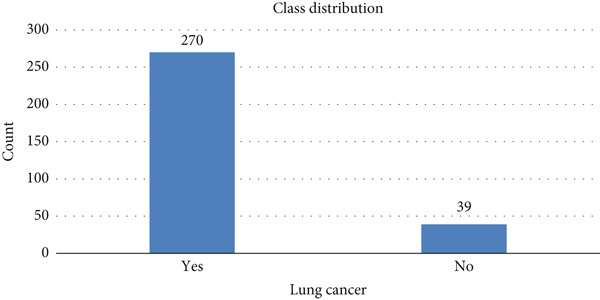
Class “lung cancer” count.

The target “lung cancer” is represented in Figure 10. The class distribution shows that class “yes” has a count of 270 and class “no” has a class count of 39.

**Figure 11:**
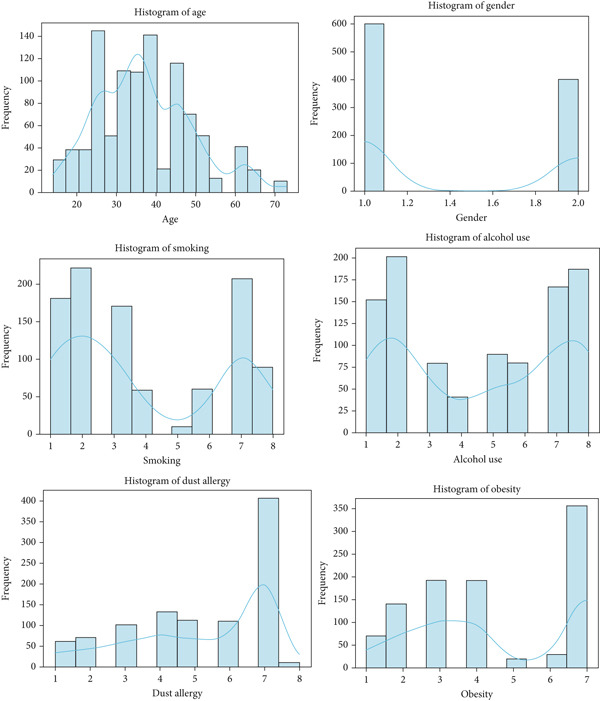
Histogram of cancer patient dataset.

Data visualization is crucial to display the features concerning the disease that are aimed at classification [71]. Histograms are a way to visualize the distribution of a dataset′s attributes [72]. A histogram with density curves for the cancer patient dataset is shown in Figure 11.

**Figure 12:**
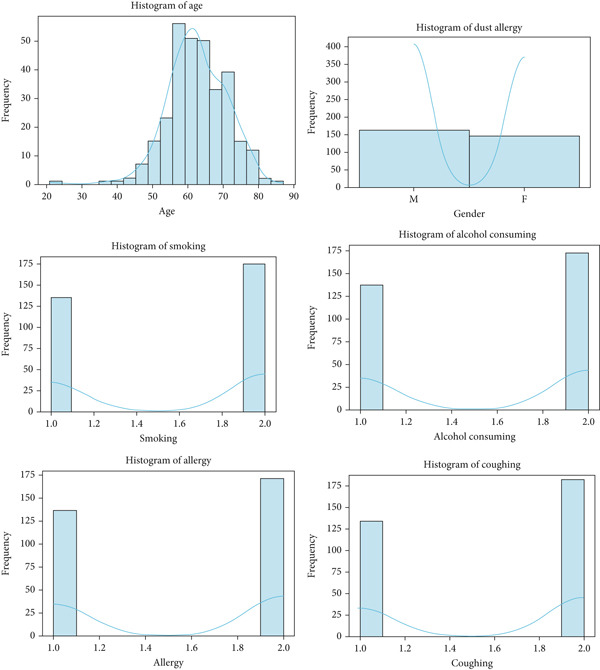
Histogram of survey cancer dataset.

Figure 12 shows a varied age distribution, while other attributes demonstrate a normal distribution with two peaks in the survey dataset.

**Figure 13:**
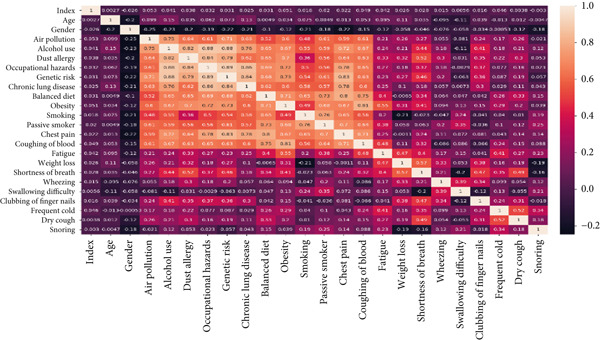
Heatmap of cancer patient dataset.

Heatmaps effectively visualize data using colors to indicate the intensity of specific attributes as provided in Figure 13.

**Figure 14:**
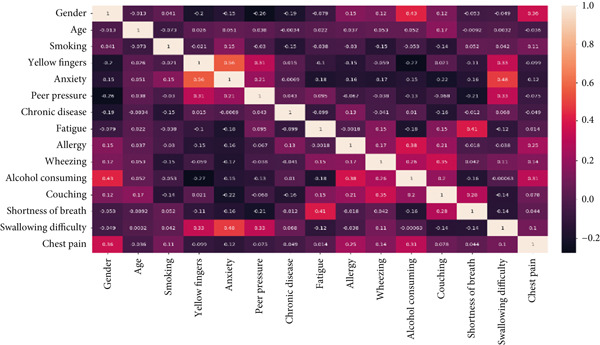
Heatmap of survey cancer dataset.

The distribution and intensity of the attributes of the survey cancer dataset are provided using a heatmap. It displays the weightage of features on the basis of correlated values. A scale is provided, which indicates the mapping of colors according to the ranges from 0.2 to 1.0, as given in Figure 14.

Boxplots of age show the absence of outliers as whiskers extend to the minimum and maximum limits, with no values outside of the plot as given in Figure 15.

Figure 15Box plot of age.

**Figure:**
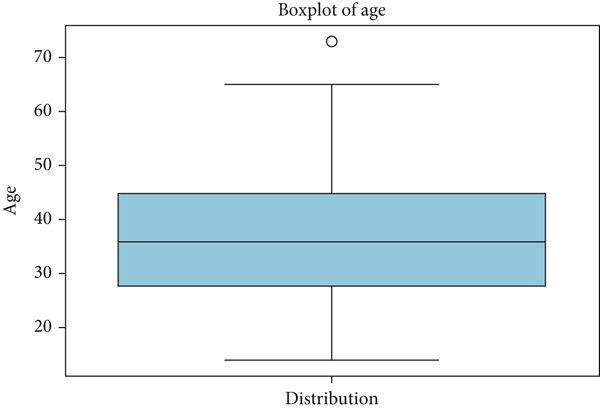
(a) Age: cancer patient dataset

**Figure:**
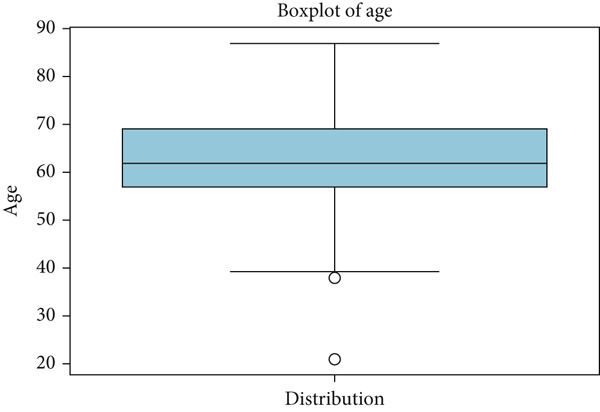
(b) Age: survey cancer dataset

### 3.2. Data Preprocessing

Preprocessing is crucial for data quality and efficiency, especially in oncology [73]. We performed cleaning, transforming, and preparing data [74]. During transformation, data were encoded into categorical variables, and feature values were converted to a numeric format using label encoding [75]. It was done by using the label encoding method. Detailed explanations for each step follow.

#### 3.2.1. Handling Missing Values

Exhaustive clinical information is required for diagnostic decision‐making, which is why missing data is important [76]. isnull() was used to ensure a complete and intact dataset. Duplicate values were also checked.

#### 3.2.2. Data Sampling

The SMOTE is a potent oversampling method for handling data imbalance [77]. It yields artificial data points by bridging the gaps between a data point in the minority class and its nearest neighbors using linear interpolation. It produces synthetic minority class samples to handle the imbalanced classes [78]. In this work, SMOTE sampling has been used to handle imbalanced classes. After applying the SMOTE sampling technique, the samples obtained for the datasets are equally distributed, as shown in Figure 16.

Figure 16After SMOTE sampling. (a) Cancer patient dataset after SMOTE. (b) Survey lung cancer after SMOTE.

**Figure:**
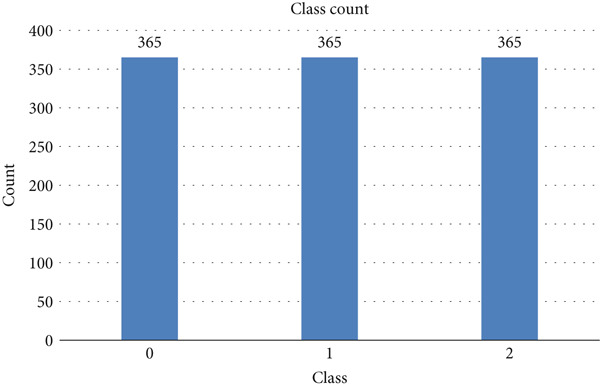
(a)

**Figure:**
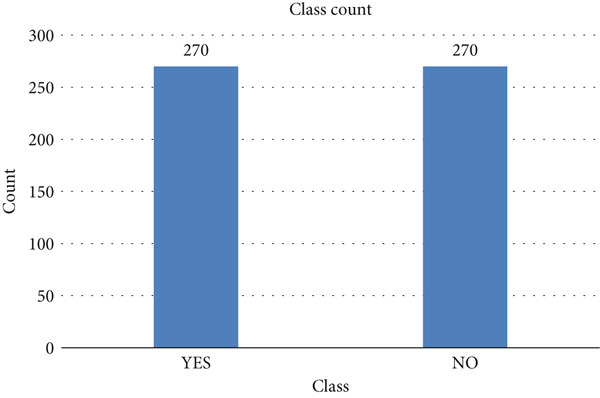
(b)

#### 3.2.3. Experimentation for Feature Selection

In machine learning, feature selection is crucial because most datasets contain irrelevant, noisy, or highly correlated attributes. It helps eliminate unnecessary attributes with minimal loss of information. Various filter methods, such as SelectKBest, rank features based on statistical measures or scores.

ANOVA is effective for multiclass classification tasks. It compares group means to test the null hypothesis that samples come from the same population distribution [79]. The *F*‐statistic calculates the variance ratio to rank features. The decision rule determines the test outcome [80]. For feature selection, the KBest algorithm with the f_classif function performs an ANOVA test to compute *F*‐values and *p* values, with SelectKBest selecting top features based on these scores.

(1) ANOVA=F‐value between class/feature.

Nine features selected by this method are given in Table 10.

**Table 10:** Feature selection of the datasets.

**Dataset 1: Cancer patient dataset**		**Dataset 2: Survey cancer dataset**	
**Air pollution**	**Balanced diet**	**Yellowing fingers**	**Alcohol consumption**
Alcohol consumption	Obesity	Peer pressure	Cough
Genetic risk	Allergy	Fatigue	Difficulty swallowing
Workplace hazards	Passive smoking	Allergy	Chest pain
Blood in cough		Wheezing	

The dataset was split into training and testing sets, with *K*‐fold cross‐validation applied to prevent overfitting and maintain class distribution. SHAP analysis on the model is shown in Figure 17.

Figure 17SHAP analysis on (a) Dataset 1 and (b) Dataset 2.

**Figure:**
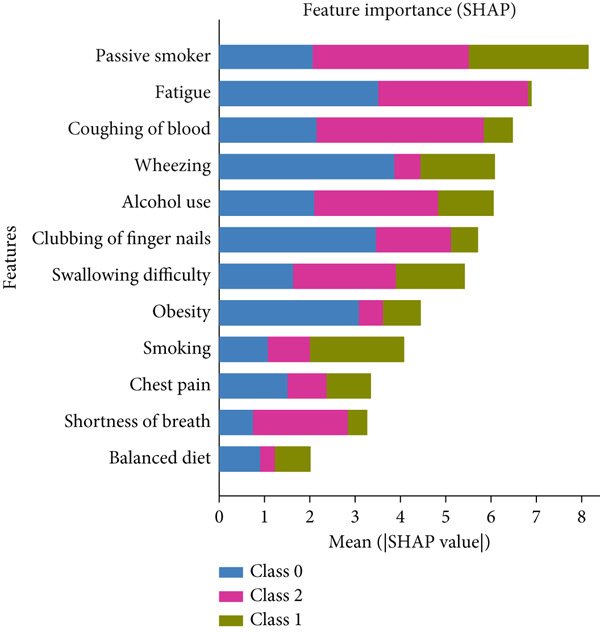
(a)

**Figure:**
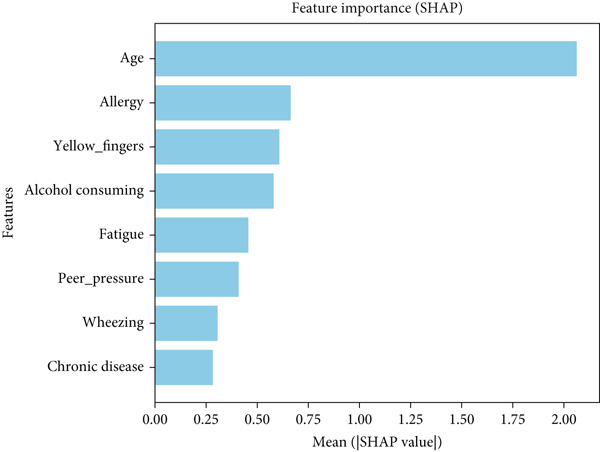
(b)

The SHAP results from both datasets show that a mix of smoking habits, respiratory symptoms, and lifestyle factors shapes lung health. Passive smoking, wheezing, alcohol use, and coughing of blood stood out in one dataset, while age, yellow fingers, allergies, fatigue, and chronic disease were more important in the other. The findings suggest that smoking‐related risks and breathing issues are key warning signs, but age and overall lifestyle still play a big role in predicting lung disease.

### 3.3. Experiments With Various Classifiers

The experiments have been done using various machine learning algorithms on the datasets. The classifiers were chosen from Table 7. The high‐performing classifiers were then combined using an ensemble. A 10‐fold cross‐validation is used to ensure that the model is not overfit. The algorithms used in the experiments are described in detail.

### 3.4. DT

The DT is a tree‐based model that assigns the data values to the actual label class using decision rules. According to the rules, the split of the population occurs in uniform sets [81].

### 3.5. LR

LR forecasts the likelihood of the occurrence of a label class. LR can be applied to binary and multiclass datasets, as it has binary and multiclass functions [82]. Previous lung cancer prediction models have used LR [83]. On the basis of these probabilities, the data points are labeled with a fixed threshold. The LR formula is as follows.

(2) πx=eβ01101+∑ni=βixi+eβ+∑ni=βixi.

Here, *π*(*x*) represents the expected outcome with respect to the value provided for independent variables *E*(*Y*|*X*). The probability of occurrence of the label class is predicted by fitting the data to the logit function, hence called logit regression [84]. The logistic function is a mathematical S‐shaped sigmoid function as given by the equation as follows:

(3) fx=11−exp−z.

### 3.6. RF

RF classifier is a set of predictor trees. The value of each tree in the set is based on a randomly sampled random vector [85]. It is an ensemble learning method consisting of multiple DTs for the purpose of classification [86]. For RFC, the number of estimators was set to 100, the entropy criterion, and 10 max_depth.

### 3.7. KNNs

KNN is a popular supervised learning algorithm that divides a given dataset into various clusters [87]. It is an adaptive classifier and can be applied to classification problems. It works by grouping the items that are similar by calculating the distance between different data points. It is classified on the basis of the majority of its nearest neighbors [88].

### 3.8. NB

NB works on the principles of Bayes′ theorem, which has conditional probability. It is given by the following formula:

(4) PX=number of favorable outcometotal number of possible outcome.

Here, *P*(*X*) refers to the probability of *X* events occurring, concerning the number of favorable outcomes and the total number of possible outcomes [89].

### 3.9. SVM

SVM classifies the data by plotting the raw data as dots in *n*‐dimensions. The value of *n* is the representation of the number of attributes [90]. It is suitable for categorizing different types of carcinomas due to its grouping technique [91].

### 3.10. Ensemble Voting Classifier

The ensemble is an adaptive method that works as a single classifier by integrating a group of different algorithms. The ensemble models are in practice as an existing alternative because they are observed to achieve a high accuracy rate for healthcare applications [92]. A type of computational ensemble is a voting classifier. It predicts the outcome of the target based on the most votes in favor after combining the individual outcomes of each algorithm. The main idea is to build one ensemble model instead of various classifiers to enhance the performance [93]. Cross‐validation is used, and a subclassifier is obtained using the voting method [94]. In this work, hard voting is used, which predicts the target class with majority votes [95]. The dataset is split and used to train the model with classifiers. Then, it proceeded to validation. The unused test set is processed during validation to get a high accuracy rate for the classifiers [96]. The model was implemented with fixed random_state = 42, and key hyperparameters (e.g., SMOTE *k* = 5, SVM kernel = rbf, *C* = 1.0, *γ* = scale) were explicitly defined to ensure reproducibility. A stratified and nested cross‐validation pipeline was used, with SMOTE applied only to training folds to prevent data leakage.

## 4. Results

Machine learning offers a variety of parameters to determine the outcome of the model, including *F*1 measure, precision, and accuracy [97]. For classification models, the performance evaluation metrics include accuracy, *F*1 measure, sensitivity, and precision [98]. The performance of the classifiers was checked using the evaluation metrics. Accuracy is the ratio of accurate predictions to incorrect predictions [99]. This is given mathematically as follows:

(5) Accuracy=TrueNegative+TruePositiveTrueNegative+FalseNegative+FalsePositive+TruePositive.

The probability ratio predicts the duration of outcome changes affecting disease likelihood, based on sensitivity and precision [100]. Precision (*p* value) is the percentage of true positives (TP) among predicted positives, while the *n*‐value represents the complement. The formulas for *p* value and *n*‐value are shown in the following:

(6) p value=True PositiveFalse Positive+True Positive.

(7) n value=True NegativeTrue Negative+False Negative .

Sensitivity is the rate at which a model predicts future instances. It is mathematically represented as follows:

(8) Sensitivity=True PositiveFalse Negative+True Positive .

When the number of FNs and FPs varies, the *F*1‐measure, which is the average of precision and recall, can be calculated [101]. It is demonstrated mathematically in (9).

(9) F1 measure=2×Precision×RecallPrecision+Recall.

Performance of the algorithms applied to the cancer patient dataset is given in Table 11.

**Table 11:** Performance metrics for cancer patient dataset.

**Classifiers**	**Accuracy (cancer patient dataset)**	**Accuracy (survey cancer dataset)**
Decision tree	96%	89%
K‐nearest neighbors	79%	86.1%
Logistic regression	94.0%	94.0%
Naïve Bayes	57.5%	89%
Proposed model	99%	92.5%

**Figure 18:**
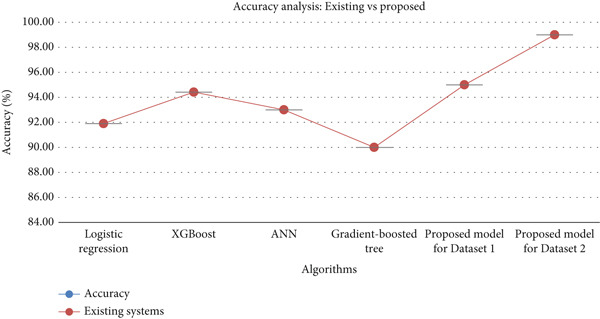
Accuracy comparison.

The proposed model outperforms the other classifiers, showing 99% on the cancer patient dataset across all metrics. DT and LR perform well; however, KNN and NB obtain lower values. KNN and NB show lower performance rates than DT and LR on the survey cancer dataset. The performance metrics clearly show that the proposed model is significantly better than the other classifiers with 92.5% accuracy, 94% for recall, precision, and *F*1 score. An overall comparison analysis of the accuracy rate obtained by some of the existing systems on the selected dataset is illustrated in Figure 18.

**Figure 19:**
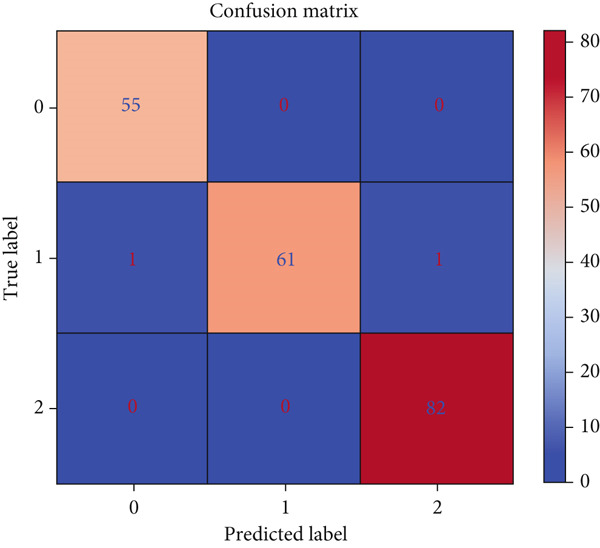
Confusion matrix of cancer patient dataset.

The model exceeded existing ones using classifiers such as LR, XGBoost, ANN, and gradient‐boosted tree. The confusion matrix shows TPs, true negatives (TNs), FNs, and FP, analyzing strengths and weaknesses [102]. It serves as a performance metric [103] holding actual and predicted values [104]. Performance metrics across 10‐fold cross‐validation were accuracy: 0.991 ± 0.009, *F*1 macro: 0.991 ± 0.010, precision macro: 0.991 ± 0.009, and recall macro: 0.991 ± 0.010. Figure 19 shows the confusion matrix, indicating high true values and minimal false values, reflecting strong predictive capability.

**Figure 20:**
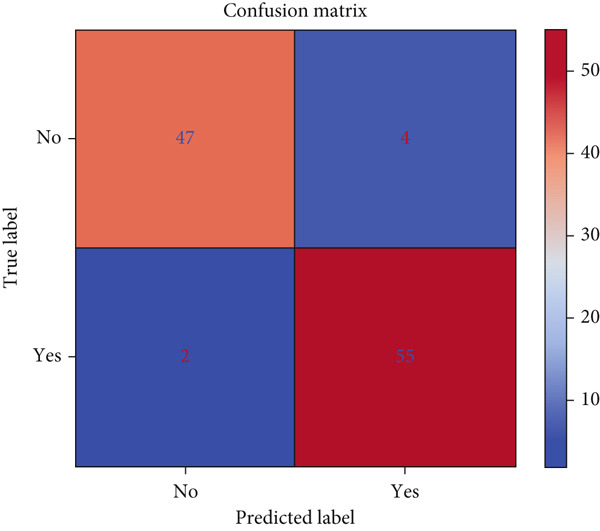
Confusion matrix of survey cancer dataset.

Similarly, Figure 20 highlights high TP and TN with minimal misclassifications. The model achieved accuracy: 0.894 ± 0.050, *F*1 macro: 0.763 ± 0.090, precision macro: 0.815 ± 0.129, and recall macro:0.782 ± 0.117, with a final test set accuracy of 0.925.

## 5. Discussion

This research presents a systematic review and a framework for effective lung cancer prediction using machine learning. Research gaps are identified, and a framework to address them is proposed. We followed five machine learning steps, from data gathering to performance evaluation, and introduced a high‐performing framework. A comparison with the existing systems is given in Table 12.

**Table 12:** Comparison with existing studies.

**Author**	**Year**	**Dataset**	**Data imbalance**	**Feature selection**	**Features**	**Algorithm**	**Validation**	**Accuracy**
Abdullah et al. [45]	2021	UCI (Kaggle)	—	Correlation method	—	SVM	—	95.56%
Mamun et al. [46]	2022	UCI (Kaggle)	—	—	—	XGBoost	Cross‐validation	94.42%
Vieira et al. [47]	2021	UCI (Kaggle)	—	Information gain, chi‐square, Gini index, and gain ratio	—	ANN	Split validation	93%
Faisal et al. [48]	2018	UCI (Kaggle)	—	—	—	Gradient‐boosted tree	Cross‐validation	90%
Radhika et al. [49]	2019	UCI (Kaggle)	—	—	—	Logistic regression	Cross‐validation (folds = 7)	96.9%
Viji Cripsy and Divya [50]	2023	UCI (Kaggle)	—	PCA with Ranker method	—	Logistic regression	—	91.90%
Dritsas and Trigka [51]	2022	UCI (Kaggle)	—	—	—	SVM	Cross‐validation	95.4%
Proposed model	2025	Kaggle	SMOTE	ANOVA	9	LR, SVM, and RF via voting ensemble	Cross‐validation	Dataset 1 = 99*%* Dataset 2 = 92.5*%*

The research questions addressed in Section 4 highlight how classifiers track patterns to accurately predict based on their learning methods. Performance varies by computational measures and factors. Several effective classifiers from the literature were noted, though AI algorithms have limitations. AI′s ability to adapt through learning and uncover patterns far outweighs these challenges [105], making it an invaluable tool in advancing lung cancer detection. In this study, two independent datasets were utilized, addressing missing values and using SMOTE to reduce class imbalance. ANOVA was applied for feature selection, and an ensemble of LR, SVM, and RF achieved a strong performance. To make the results interpretable, SHAP analysis was used, which provided clear insights into feature importance.

## 6. Conclusion and Future Work

Lung cancer is a fatal disease. With machine learning, the identification of lung cancer has made significant advancements, providing invaluable support for practitioners. In this paper, a comprehensive literature review on lung cancer prediction using machine learning was conducted to answer the four research questions raised. In the SLR, 40 papers were selected using the tollgate approach and quality assessment criteria. The answers based on the literature review compared and analyzed the classifiers, significant features, and traditional practices with the machine learning model. It also suggested the potential pros and cons of the current research. A machine learning framework has been proposed for accurately predicting lung cancer using two datasets from Kaggle. Machine learning processes were applied to the chosen datasets and outcomes were evaluated. The voting ensemble with SVM, RF, and LR with 10‐fold cross‐validation has shown 99% accuracy on the cancer patient dataset and 92.5% accuracy on the survey cancer dataset. While the proposed framework achieves competitive performance, we acknowledge that this work represents an incremental benchmarking effort. The application of machine learning serves as a valuable addition to traditional practices. Integration of different types of datasets can be used to apply machine learning techniques to analyze the outcomes further. The framework highlights patient‐specific risk factors such as age, lifestyle habits, and comorbidities, making the results more interpretable for healthcare professionals. The proposed model could be deployed as a decision‐support tool to assist in early screening and risk assessment, complementing clinical judgment. However, this research is based on publicly available Kaggle datasets. The datasets had multivariate and binary classes with significant attributes that helped in predicting early‐stage lung cancer with accurate results. While SMOTE helps address class imbalance, its synthetic nature can inflate performance, especially on small datasets. Furthermore, the small dataset size may limit generalizability. In future work, we aim to validate our framework on more diverse, larger, and clinically annotated datasets to strengthen its generalizability.

## Ethics Statement

This study uses publicly available, anonymized data; it did not involve any human participants, animals, or patient intervention directly.

## Conflicts of Interest

The authors declare no conflicts of interest.

## Funding

The research was carried out with the support of GUST, and the APC of this article was funded by the Joint Information Systems Committee (10.13039/501100000821).

## Data Availability

Datasets used in this paper are publicly available. The code supporting the findings of this study is available at https://github.com/Azkamir/lungCancer_model.
